# *Eimeria* infections of plateau pika altered the patterns of temporal alterations in gut bacterial communities

**DOI:** 10.3389/fmicb.2023.1301480

**Published:** 2024-01-11

**Authors:** Maoping Li, Suqin Wang, Liang Zhong, Petr Heděnec, Zhaoxian Tan, Rong Wang, Xinyang Chen, Yan Zhang, Bingmin Tang, Huakun Zhou, Jiapeng Qu

**Affiliations:** ^1^Sanjiangyuan Grassland Ecosystem National Observation and Research Station, Key Laboratory of Adaptation and Evolution of Plateau Biota, Northwest Institute of Plateau Biology, Chinese Academy of Sciences, Xining, China; ^2^Qinghai Province Key Laboratory of Animal Ecological Genomics, Xining, China; ^3^University of Chinese Academy of Sciences, Beijing, China; ^4^Institute for Tropical Biodiversity and Sustainable Development, University Malaysia Terengganu, Kuala Terengganu, Terengganu, Malaysia; ^5^School of Life Science, Qinghai Normal University, Xining, China; ^6^Grassland Station of Qinghai Province, Xining, China

**Keywords:** *Eimeria* infection, gut bacterial community, plateau pika, temporal alteration, host response

## Abstract

Intestinal parasites, such as *Eimeria*, are common among plateau pika (*Ochotona curzoniae*). The gut microbiome is an essential driver of the host response to gastrointestinal parasites. However, the effects of intestinal protozoal parasites on the temporal variations in the gut microbiome and behavioral and physiological activities remain unknown. Our study conducted treatments involving experimental feeding of pika with *Eimeria* oocysts or anticoccidia under laboratory conditions to focus on the parasite-associated alterations in gut bacterial communities, host behavioral activity, physiology, and host–bacteria relationships. The results showed insignificant differences in bacterial community structures among treatments on the basis of Bray–Curtis distance metrics, whereas the patterns of temporal alterations in the bacterial communities were changed by the treatments. Bacterial alpha diversities did not vary with the treatments, and experimental feeding with *Eimeria* slowed down the decrement rate of alpha diversity. Furthermore, few bacterial members were significantly changed by the treatments—only the genus *Ruminococcus* and the species *Ruminococcus flavefaciens*, which were associated with energy metabolism. Experimental feeding with *Eimeria* modified the temporal variations in the bacterial members, including a lower loss rate of the relative abundance of the dominant families Muribaculaceae and Ruminococcaceae in the group with *Eimeria* experimental feeding. Moreover, a shifting energy trade-off was suggested by the parasite-induced increments in thyroid hormones (triiodothyronine and tetraiodothyronine) and decrements in exploration behavior in the group with *Eimeria* feeding. However, we did not detect specific connections between gut bacterial communities and pika behaviors and physiology in terms of energy trade-offs. Further in-depth research is needed to examine the role of *Eimeria*-modified differences in the gut bacteria of plateau pika.

## 1 Introduction

The digestion tract hosts a large number of microorganisms that can regulate animal metabolism, nutrition, immunity, behavior, and physiology (Tremaroli and Bäckhed, 2012; Brosschot and Reynolds, 2018; Carrasco et al., 2019; Madlala et al., 2021). Changes in gut microbial community composition are likely to be interconnected with functional changes in gut microbiomes, which in turn alter the host’s health (Wang et al., 2022). Most of the changes in gut microbiomes are host responses to the effects of various factors, and these changes commonly affect host health (Hauck, 2017).

The digestive tract of wild animals is commonly infected by parasites in nature (Gulland, 1995). Gut microbial communities are changed by parasitic infections (Lee et al., 2014; Kreisinger et al., 2015; Trzebny et al., 2023). For example, mice had an increased abundance of gut microbiota and a high relative abundance of *Bacteroides* in the intestine after being infected with the nematode parasite *Trichinella spiralis* (Xing, 2017). The gut microbial diversities modified by parasitic infections can be decreased (Cattadori et al., 2016), unchanged (McKenney et al., 2015), or increased (Broadhurst et al., 2012). Meanwhile, microbial compositions commonly change after parasitic infection (Paris et al., 2020). For instance, the relative abundance of S24-7 (Bacteroidetes) is increased by the presence of parasitic tapeworms in *Apodemus flavicollis* (Kreisinger et al., 2015). Gut parasites generally interact with the gut microbiota, and such interactions may alter gut microbial communities (Glendinning et al., 2014). The interactions include food nutrient competition, which reduces the available nutrients for microorganisms and animal hosts (Stensvold and Giezen, 2018), thus altering the intestinal microbial communities (Trzebny et al., 2023). In addition, intestinal parasites can disrupt gut metabolites, alter the physico-chemical characteristics of the intestinal environment, and affect the quantity and structure of the gut microbiota (Wang et al., 2023).

Gut parasites affect the structure and function of the gut microbiota, which in turn influences the behavior and physiology of the host (Stensvold and Giezen, 2018; Madlala et al., 2021). Parasite-associated variations in gut microbial communities are followed by shifting interactions among gut microbiota, parasites, and the host, with the host experiencing harmful, neutral, or beneficial effects (Hauck, 2017; Cortés et al., 2020b; Drew et al., 2021). Shifts in gut microbial communities may favor the growth of other pathogens (Antonissen et al., 2016), increase resistance against infection damage (Brosschot and Reynolds, 2018; Gao et al., 2020), or enhance the persistence of parasitic infection (Reynolds et al., 2014). Therefore, the relationships among parasites, animals, and gut microbiota are complex. Studying the relationship among wildlife, parasites, and gut microbiota is crucial for understanding species coexistence.

Plateau pika (*Ochotona curzoniae*) is a keystone species on the grassland of the Qinghai–Tibetan Plateau, which is widely distributed in the Qinghai–Tibetan Plateau (Li H. et al., 2016; Yu et al., 2022). Pikas have many ecological functions in grassland ecosystems (Smith et al., 2019). The population density of pikas is closely correlated with grassland ecosystems (Wei et al., 2023). For example, an appropriate population density of pikas may increase vegetation diversity, promote soil water infiltration, and provide food sources for carnivorous animals (Smith and Foggin, 1999). Specifically, when the density of pikas is between 20 and 42 burrows/mu, plant diversity is improved by pika disturbance (Pang and Guo, 2018). However, a population density that is too high can damage ecosystems and lead to grassland degradation (Zhao et al., 2020). For instance, when the density of pikas exceeds 470 burrows/mu, soil nutrients, such as nitrogen and phosphorus, are reduced, and ecosystem function is damaged (Yu et al., 2017). At present, in many sites on the Qinghai–Tibetan Plateau, the population density of pikas is too high, resulting in a severe ecological problem.

*Eimeria* is a common parasite in the intestines of pikas, and *Eimeria* infection may lead to the death of pikas (Bian et al., 2011; Du et al., 2012). Therefore, *Eimeria* may be a potential ecological control agent for controlling plateau pika. Although many studies have been conducted on the relationship between the dose of *Eimeria* and the mortality rate of plateau pikas, whether *Eimeria* infection affects the gut microbiota, physiology, and behavior of plateau pikas remains unclear. Studying this issue can help in understanding the inherent relationships between parasitic infections and hosts.

In this study, we used plateau pikas as a model and performed high-throughput sequencing and behavioral and physiological tests to explore the mechanism of the effects of *Eimeria* infections on the intestinal microbes, behavior, and physiology of wild animals. The aim was to elucidate the internal mechanism of the effect of parasitic infections on animals and provide a scientific basis for further development of biological control technologies for plateau pikas. We hypothesized that experimental feeding with *Eimeria* considerably changes the gut bacterial community, the behavioral, physiological, and metabolic characteristics, and their relationships. We aim to answer the following questions: (1) How do the behavioral, physiological, and metabolic characteristics and gut bacterial communities of pikas vary with *Eimeria*? (2) Do the relationships between gut bacteria and pika behavioral, physiological, and metabolic characteristics vary among treatments? (3) What is the role of these variations in the parasites or host?

## 2 Materials and methods

### 2.1 Capture and treatment of wild plateau pikas

The experimental site for capturing wild animals is located in Menyuan Hui Autonomous County, Haibei Tibetan Autonomous Prefecture, Qinghai Province (37°56′N, 101°41′E; 3,062 m). The region has a plateau continental climate, with cold and dry winters and cool and humid summers. The average daily temperature difference is 11.6°C–17.5°C, and the annual sunshine duration is 2,232–2,741 h (Mi and Zhang, 2016).

In September 2020, 30 healthy adult plateau pikas weighing over 130 g were live captured using the string trap method. The pikas were brought back to the laboratory, and each pika was raised in a single cage (450 mm × 289 mm × 180 mm) in the animal feeding room of the Northwest Institute of Plateau Biology, Chinese Academy of Sciences. The bottom of the cage was covered with sawdust. With reference to the study of Bian et al. (2011), the cage was washed daily and scalded with boiling water, and sufficient water and standard rabbit pellet feed were provided (Beijing Ke’ao Xieli Feed Co., Ltd.). Room temperature and photoperiod were the temperature and illumination of the natural environment, respectively.

### 2.2 Experimental design

Coccidia oocysts were isolated from the intestinal content mixture of several plateau pikas that died of coccidiosis. We collected a 2 g mixture and added it to 5 mL of a 0.8% sodium chloride solution. After thorough grinding, the mixture was centrifuged at a speed of 3,000 rpm for 5 min. The sediment was isolated and collected as oocyst fluid. The isolated coccidia oocysts were stored in a 2.5% potassium dichromate solution and sporulated in a constant-temperature incubator at 27°C. *Eimeria* oocysts were obtained after the proliferation of multiple egg sacs. The oocysts were mixtures of *Eimeria* species that were isolated from the intestinal contents of the dead plateau pikas. In this experiment, sodium sulfaclopyrazine was used as a drug to suppress coccidia. Sodium sulfaclopyrazine can compete with cyanobenzoic acid for dihydrofolate synthetase, impede dihydrofolate synthesis, and ultimately affect the synthesis of nuclear proteins, thus inhibiting the growth and reproduction of coccidia (Zhang et al., 2012). The anticoccidial index (ACI) was used as a drug efficacy indicator. The judgment criteria were as follows: ACI ≤ 120 was invalid, 120 < ACI ≤ 160 denoted low efficiency, 160 < ACI ≤ 180 indicated medium efficiency, and ACI > 180 was considered to be highly effective. Previous experiments on using sulfaclopyrazine sodium for the prevention and treatment of intestinal coccidia have shown that ACI is 147–197, which indicates a good effect on repelling coccidia (Xue et al., 2012).

After 1 month of laboratory domestication, 30 plateau pikas were randomly divided into three groups. Each group consisted of 10 individuals. The first group was fed with 20× effects of 10^5^ *Eimeria*/mL and labeled as Group PA+. The second group was fed with sulfaclopyrazine sodium as an equivalent of ACI (the ratio was 0.0012× body mass of pika, and physiological saline was added to 1 mL) and labeled as Group PA−. The third group was fed with 1 mL of normal saline and labeled as Group C (control). Previous studies have shown that the schizogenesis of *Eimeria* mainly destroys the intestinal tissue of the host (Cai et al., 2001).

In accordance with the pathogenicity cycle of *Eimeria*, experimental data were collected at four time points, that is, days 0, 5, 8, and 18 of the experiment. The behavior (exploration activity), physiological characteristics (cortisol, triiodothyronine [T3], tetraiodothyronine [T4], resting metabolic rate, and weight), *Eimeria* oocyst number, and intestinal microorganisms of the plateau pikas were measured at each time point for each experimental individual.

The cages were washed daily and scalded with boiling water to prevent cross-contamination by oocysts. At 7:00 a.m. on each test day, the leftovers from the day before were collected, and 50 g of new feed was placed in each cage. Sterile tweezers were used to collect 6 g of fresh feces from each cage, and the feces were divided into five packages. Five parts were placed in a sterile frozen storage tube, which was marked afterward. The first four samples were stored in a refrigerator at −80°C, and the fifth sample was stored in a refrigerator at 4°C. The samples were used for the determination of cortisol, T3 and T4 contents, and the numbers of coccidian oocysts and intestinal microorganisms. Subsequently, the behavior and physiological parameters, including exploration activity, body mass, and resting metabolic rate, of the plateau pikas were measured.

### 2.3 Determination of the number of *Eimeria* oocysts

McMaster’s method was utilized to count the number of coccidia oocysts in the feces (REF). A total of 2 g of feces was added to 20 mL of saturated saline, mixed well, and filtered through 40- and 100-mesh filter sieves. Exactly 1 mL of the filtrate was taken and added to 9 mL of water. After fully mixing the diluent, a capillary pipette was used to suck out a small amount of liquid. The liquid was dropped into the counting chamber of the McMaster counter, which was then placed on a microscope table. After a few minutes, we used a low-power microscope to count all the coccidian oocysts in the two counting chambers. The average value was obtained and converted into the number of coccidian oocysts per gram of feces.

### 2.4 Physiological trait measurement

The resting metabolic rate (RMR) of the plateau pikas was measured using a portable animal respiratory metabolic measurement system. The animals were placed in a transparent breathing chamber located in a thermostat. The heat-neutral zone of pika is in the temperature range of 25°C–30°C (Wang et al., 1993), within which the minimum basic or static metabolic rate level is maintained (Bligh and Johnson, 1974). The thermostat temperature was set to 27°C. The sampling interval for each channel was set to 3 min, and the airflow rate from the air pumped into the breathing chamber was set to 600 mL/min. The first breathing chamber served as a blank control, and the three other breathing chambers were filled with pikas. The metabolic rate of the pikas was measured after 30 min of adaptation. The measurement was repeated 10 times for a total of 2 h. The determination followed the method in the literature (Otálora-Ardila et al., 2017).

### 2.5 Monitoring and analyses of behavioral activity

The exploration activity of the plateau pikas was measured using the open field test. In consideration of the activity rhythm of the plateau pikas, the exploration activity was measured from 8:00 a.m. to 11:00 a.m. The open field consisted of opaque acrylic panels with a bottom area of 50 cm × 50 cm. The center bottom (40 cm × 40 cm) was marked as the central area, and the area outside the central area was marked as the edge area. The individual to be tested was gently placed in the open field. A camera was used to record the behavior of the individual within 3 min. The EthoVision IX Animal Motion Tracking System was employed to analyze various pika behaviors, including dwell times and transit frequencies in the central and marginal areas (Qu et al., 2018, 2019). Exploration activity was determined based on the time that the animals stayed in the central area of the open field and the number of times they crossed the center. After each measurement, 75% alcohol was used to wipe the open field and clean up the residual feces, urine, and hair to avoid affecting the subsequent measurements.

### 2.6 Fecal extraction and measurement of cortisol and thyroid hormones

Enzyme-linked immunosorbent assay (ELISA; Palmer et al., 2007) was used to measure the content of cortisol in the pika feces. After thoroughly grinding the pika fecal sample, 0.1 g of feces was used to prepare the extract at a 1:9 ratio. The homogenate was added to fully extract cortisol. Then, the extracting solution was placed in a 4°C freezer for 12 h and centrifuged at 3,000 r/m for 5 min at 4°C. The supernatant was obtained to test the concentration of cortisol by conducting ELISA kit testing.

Thyroid hormone (serum T3 and T4) levels are related to energy metabolism. When stimulated by external or internal factors, the concentrations of T3 and T4 increase, and the body’s energy consumption increases. On the contrary, when the secretion of T3 and T4 is too low, the metabolic rate decreases, reducing energy consumption (Eales, 1988). Thyroid hormone levels indirectly reflect energy consumption. After fully grinding the pika feces sample, 0.1 g of feces was added to a certain amount of phosphate buffer salt (PBS; pH 7.4) and quickly frozen with liquid nitrogen for storage. After the sample had melted, a certain amount of PBS (pH 7.4) was added. The homogenate was mixed thoroughly at 3,000 r/min and centrifuged at 4°C for 20 min. The supernatant fluid was collected to determine T3 and T4 using the ELISA kit.

### 2.7 DNA extraction and amplicon sequencing

A total of 120 fecal samples were collected to explore the pika gut bacterial communities. An animal fecal DNA isolation kit (DP 328, Tiangen, China) was used to extract the total sampling genomic DNA. DNA concentration and quality were assessed through spectrometry absorbance (A260/A280) using a Nanodrop 2000 spectrophotometer (Thermo Fisher Scientific, IL, United States). Polymerase chain reaction (PCR) amplification and gel extraction were performed with the methods of Li H. et al. (2016). The primers for bacteria detection were primer pairs 341F (5′-CCTACGGGNGGCWGCAG-3′) and 806R (5′-GGACTACHVGGGTATCTAAT-3′).

The V3–V4 region of 16S rDNA was amplified using specific primers with a barcode. The 16SrRNA gene of pika intestinal microorganisms was amplified using primer sequences 341F CCTACGGNGGCWGCAG and 806R GGACTACHVGGGTATCTAAT. The 25 μL PCR reaction system was a 1 μL DNA template with a pair of primers of 1 and 12.5 μL each × Taq Platinum PCR Mix and 9.5 μL of ultrapure sterile water. The PCR amplification procedure was predenatured at 94°C for 3 min, denatured at 94°C for 30 s, annealed at 55°C for 30 s, and extended at 72°C for 1 min for a total of 30 cycles. Annealing was performed at 72°C for 5 min, and the temperature was reduced to 4°C. Then, 5 μL of the reaction product was detected by electrophoresis on agarose gel with 2% concentration at 40 V for 40 min. After electrophoresis, the gel was cut and recycled, and the PCR product was purified. The purified amplification product (i.e., amplified fragment) was connected to a sequencing connector, a sequencing library was constructed, and Illumina was adopted for sequencing (Ion S5™ XL).

Low-quality reads were filtered through Usearch software, and dual-ended reads were spliced into tags. Low-quality filtering was performed on the tags, and a total of 13,545,576 high-quality clean reads were collected. Next, on the basis of the clean tags, clustering using USEARCH software was applied to remove the chimeric tags detected during the clustering process, and the abundance of operational taxonomic units (OTUs) and representative OTU sequences was determined. The dilution curve of the alpha diversity index was calculated using QIIME 1.9 software to confirm that the sequencing depth was sufficient to meet the requirements of this experiment. QIIME software was also used to calculate various alpha diversity indices, including observed species and Shannon indices, and R statistical software was employed to statistically test the alpha diversity indices of the three treatment groups. R statistical software and Bray–Curtis distance were adopted to calculate and visualize various beta diversity distances and determine the difference in the intestinal microbial structures of the plateau pikas in the various groups. The statistical testing methods included Welch’s *t*-test, the Wilcoxon rank sum test, and ADONIS. *p* < 0.05 indicated a significant difference.

### 2.8 Statistical analysis

The differences in the physiological indicators, behaviors, and hormones of the pikas in the different groups were analyzed using the one-way ANOVA. A univariate analysis of variance was also performed for the differences in intestinal microbial populations among the different groups. The relationship between time and gut microbial or physiological hormone indicators was determined via linear regression analysis. The relationship between various factors and intestinal microorganisms was analyzed using PERMANOVA with R packets. The relationship between intestinal microorganisms and various physiological and metabolic behavioral factors was analyzed using the Spearman correlation. Network analysis was performed using Gephi software, and the relationships between intestinal microorganisms and physiological and metabolic indicators in the three treatment groups were determined.

## 3 Results

### 3.1 Variations in the *Eimeria* oocyst number, behavior, and physiology of pikas

**Figure 1:**
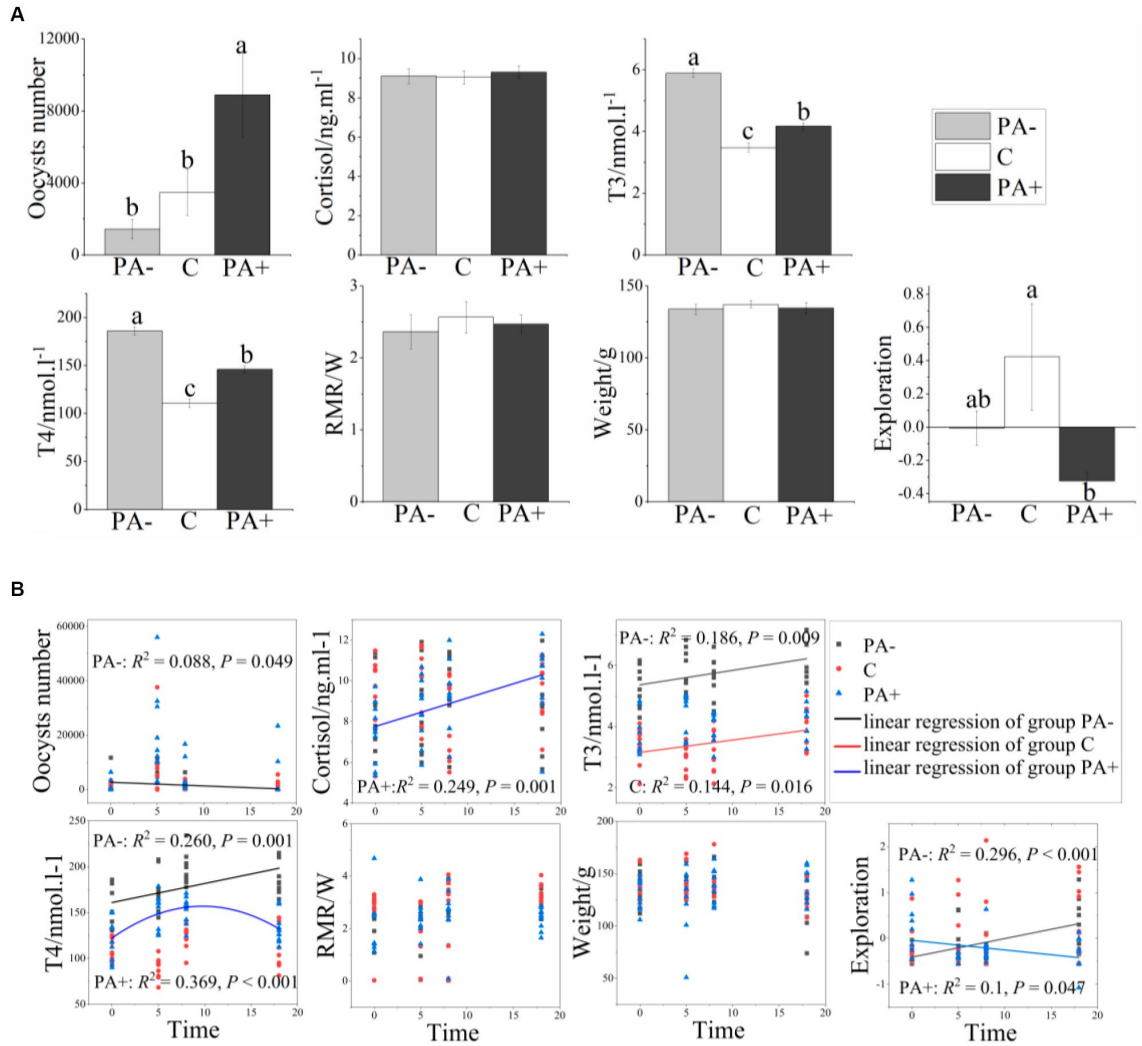
Variations in the number of *Eimeria* oocysts, physiological traits, and exploration activity of the pikas with **(A)** treatments and **(B)** raising time. Different lower-case letters indicate a significant difference at *p* < 0.05.

When the pikas were fed with *Eimeria* (Group PA+), the number of *Eimeria* oocysts and the fecal T3 and T4 contents were significantly higher than those in Group C (control group), but the exploration activity was significantly lower (*p* < 0.05; Figure 1A). The number of *Eimeria* oocysts in the group with anticoccidia (Group PA−) declined by 58.92%, and the T3 and T4 contents significantly increased compared with Group C (*p* < 0.05; Figure 1A). For Group PA+, cortisol increased significantly with time (*R*^2^ = 0.249, *p* = 0.001), whereas exploration activity declined (*R*^2^ = 0.100, *p* = 0.047). For Group PA−, exploration activity and T3 and T4 levels increased significantly as time increased, whereas oocyst number decreased significantly (Figure 1B).

### 3.2 Variations in the gut bacterial communities of pikas

With regard to the compositions of the pika gut bacterial communities, the pika gut bacterial communities were dominated by Bacteroidetes, Firmicutes, Proteobacteria, and Verrucomicrobia at the phylum level (more than 96% relative abundance; Supplementary Figure S1A). At the family level, the bacterial communities were primarily Muribaculacceae, Christensenellaceae, Akkermansiaceae, and Erysipelotrichaceae (Supplementary Figure S1B). At the genus level, the four genera with the highest relative abundance were *Christensenellaceae_R-7_group*, *Akkermansia*, *Psychrobacter*, and *Aerococcus* (Supplementary Figure S1C).

**Figure 2:**
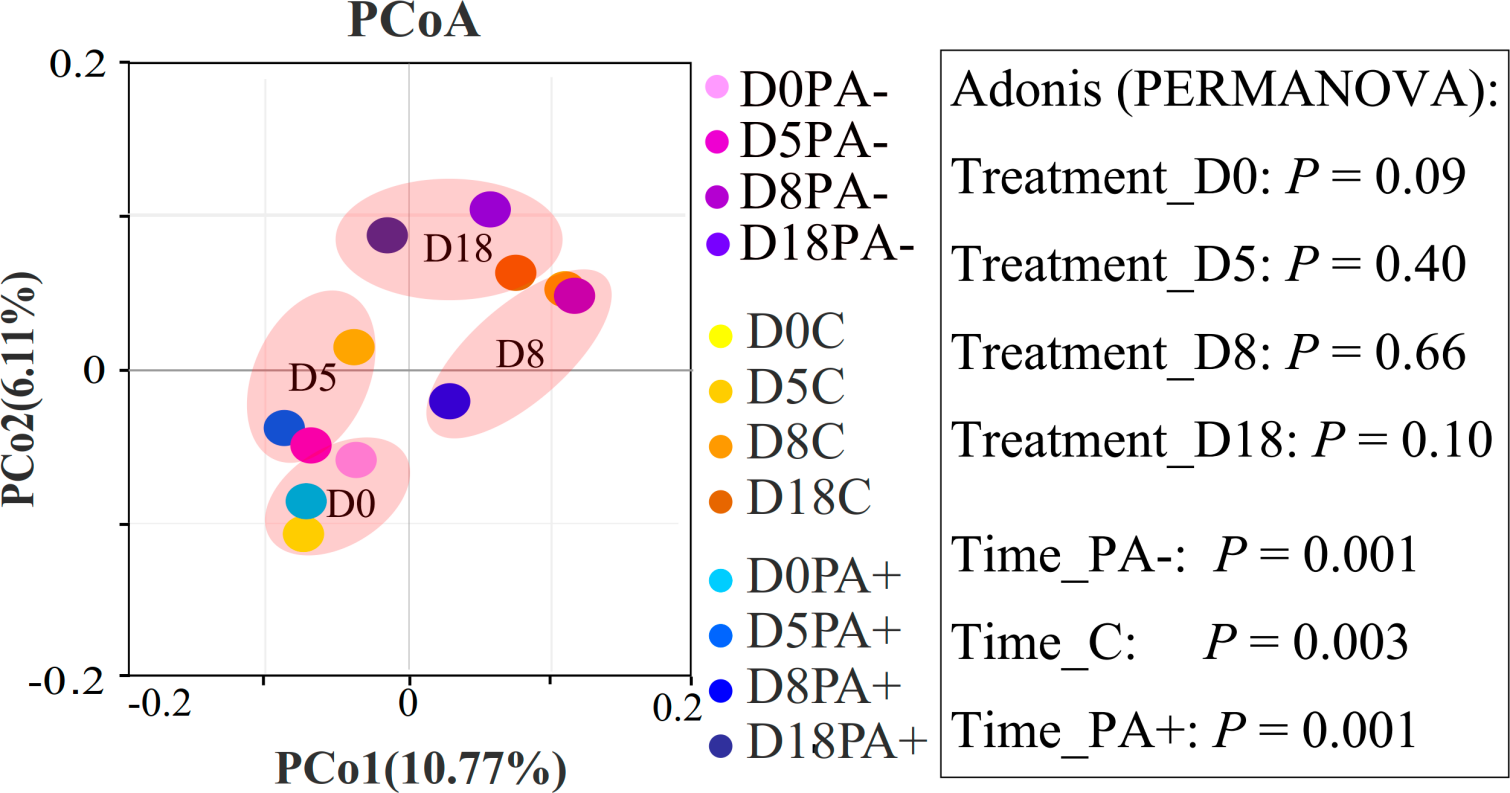
Principal coordinate analysis (PCoA) plots showing the difference in bacterial community structures between different groups based on Bray–Curtis distance metrics. The percentage of variance for each principal coordinate axis is shown in parentheses. D0 designates the experimental day 0, D5 designates the experimental day 5, D8 designates the experimental day 8, and D18 designates the experimental day 18. PA+ designates the group with feeding *Eimeria*, PA− designates the group with deworming *Eimeria*, and C designates the control group.

**Figure 3:**
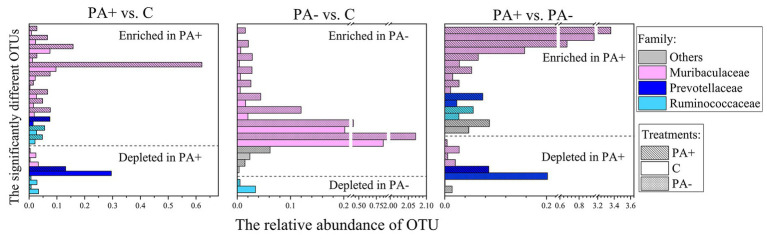
Significantly changed OTUs of pika gut bacterial communities and OTU levels in the different treatments. PA+ designates the group with feeding Eimeria, PA− designates the group with deworming Eimeria, and C designates the control group.

The bacterial community structures showed no significant difference among the treatments at each raising time stage according to the Bray–Curtis distance metrics (*p* > 0.05 based on Adonis [PERMANOVA]; Figure 2). No bacterial taxa shifted significantly among the treatment groups at phylum (mean relative abundance >0.1%) and family (mean relative abundance > 1%) levels. Meanwhile, the relative abundance of one genus (mean relative abundance > 1%), *Ruminococcus*, was significantly higher in Group PA− than in Group C and PA+ (*p* < 0.05; Supplementary Figure S2A). The relative abundance of one species (mean relative abundance > 1%), *Ruminococcus flavefaciens*, was significantly higher in Group C than in Group PA+ (*p* < 0.05; Supplementary Figure S2A). However, the changed genus and species were not significantly correlated with raising time (Supplementary Figure S2B). At the OTU (mean relative abundance > 0.01%) level, Group PA+ had more enriched OTUs compared with Groups C and PA−, and more OTUs were enriched in Group PA− compared with Group C (*p* < 0.05; Figure 3). These OTUs mainly belonged to the family Muribaculacceae. We also analyzed the variation patterns of the gut bacterial community structures along raising time by performing an indicator analysis at phylum, family, genus, species, and OTU levels. The results showed that the variation patterns differed among the various treatments (Supplementary Figures S3–S7).

**Figure 4:**
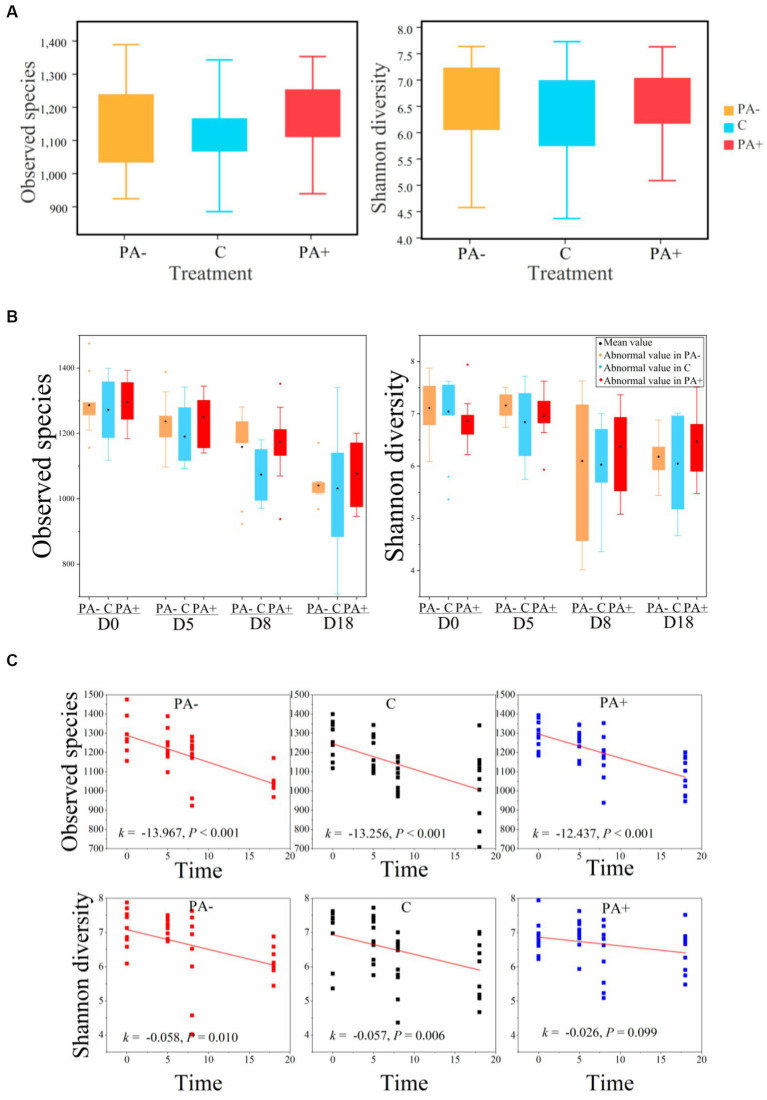
Variations in the observed species and Shannon diversity of pika gut bacterial communities **(A)** among treatments, **(B)** among groups, and **(C)** along raising time.

**Figure 5:**
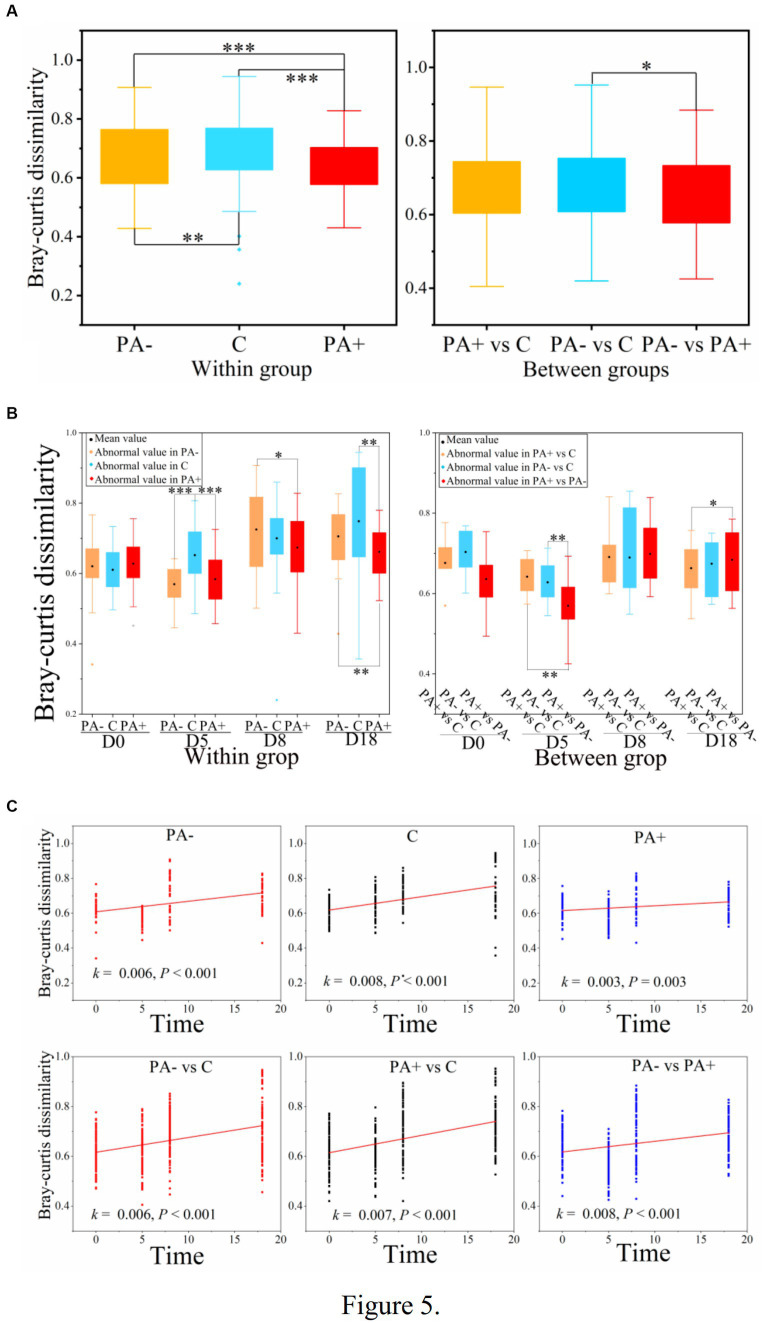
Variations in the beta diversity of pika gut bacterial communities **(A)** among treatments, **(B)** among groups, and **(C)** along raising time based on Bray–Curtis dissimilarity. One asterisk (^*^) indicates a significant difference at *p* < 0.05, two asterisks (^**^) indicate a significant difference at *p* < 0.01, and three asterisks (^***^) indicate a significant difference at *p* < 0.001.

With regard to the diversity of the gut bacterial communities, no significant differences in observed species or Shannon diversity were found among the treatment groups (*p* > 0.05; Figures 4A,B). Notably, alpha diversity (observed species and Shannon diversity) significantly declined with raising time in all the treatment groups (*p* < 0.05; Figure 4B). The decrement rate of alpha diversity was the highest in Group PA−, followed by Group C, and the lowest in Group PA+ (Figure 4C). For beta diversity, the similarity was the highest in Group PA+, followed by Group PA−, and the lowest in Group C according to the Bray–Curtis distance matrix (*p* < 0.05; Figure 5A). The within-group similarities among the treatments had no significant difference on day 0 but were significantly higher in Group PA+ than in Group C on days 5 and 18 (*p* < 0.05; Figure 5B). The within-group dissimilarity significantly increased with increasing raising time in each treatment group (*p* < 0.05; Figure 5C). The treatment with experimental feeding of *Eimeria* or anticoccidia slowed down the increased rate of within-group dissimilarity (Figure 5C). The between-group dissimilarity was significantly magnified along raising time in all the groups (*p* < 0.05; Figure 5C).

### 3.3 Variations in the correlations between gut bacterial communities and the number of *Eimeria* oocysts, behavior, and physiology of pikas

**Figure 6:**
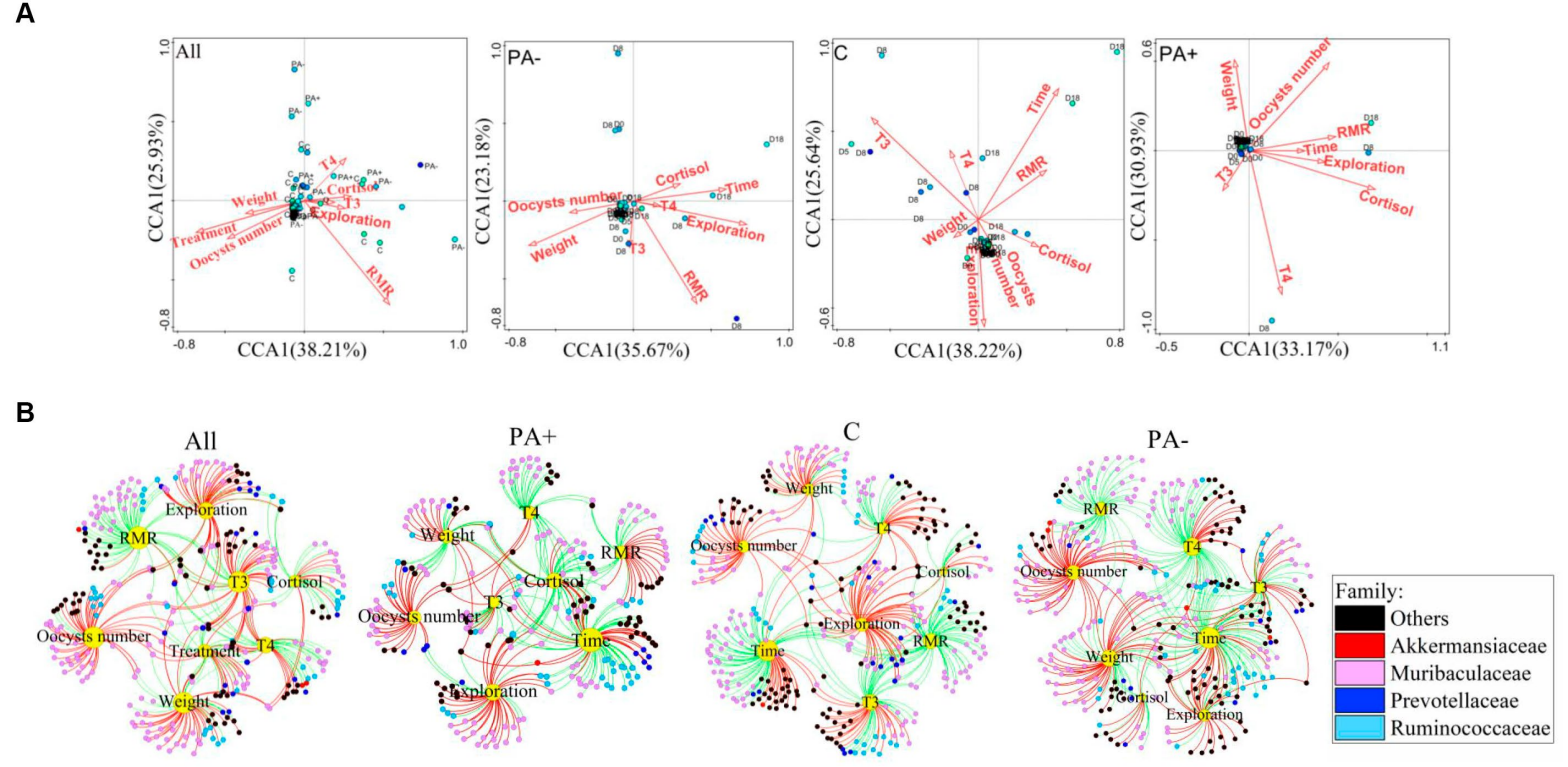
Variations in **(A)** the driving factors of pika gut bacterial communities and **(B)** significant correlations of the driving factors with the relative abundance of bacterial OTUs (*p* < 0.05). The red lines mean significantly positive correlations, and the green lines mean significantly negative correlations.

In Group C, T3, RMR, body mass, and raising time were significantly related to the bacterial communities. Oocyst number, cortisol, T4, and exploration activity were significantly correlated with the bacterial communities in Group PA+. In Group PA−, RMR, exploration activity, and increase in time were correlated with the bacterial communities (Figure 6A; Supplementary Table S1). The correlations of factors with the relative abundance of OTUs were also analyzed. Significantly positive correlations were found between oocyst number and the relative abundance of OTUs in each treatment group (*p* < 0.05; Figure 6B). The OTUs that were significantly correlated with the factors mainly belonged to the families Akkermansiaceae, Muribaculacceae, Prevotellaceae, and Ruminococceae (Figure 6B).

The relative abundance of OTUs belonging to the family Akkermansiaceae increased significantly with raising time in all the groups (Figure 6B; Supplementary Figure S8). The frequency of the significantly positive correlations between raising time and the relative abundance of OTUs belonging to Muribaculacceae was 28.57% in Group PA− and 28.07% in Group PA+; both values are much higher than the 1.85% in Group C. Furthermore, the frequency of significantly positive correlations between raising time and the relative abundance of bacterial OTUs belonging to the family Prevotellaceae was the highest in Group PA+ (87.05%), followed by Group PA− (33.33%), and the lowest in Group C (0.00%). Meanwhile, the relative abundance of OTUs belonging to Ruminococceae was significantly negatively correlated with raising time in Groups PA− and C, and 94.43% of the OTUs were negatively correlated with raising time in Group PA+ (Figure 6B; Supplementary Figure S8).

To explore the variations in the four families to which the factor-correlated OTUs mainly belonged, we analyzed the variations in the OTU number and relative abundance of the four families. The results showed that Group PA+ had more OTUs belonging to Muribaculacceae compared with Group C. Group PA− had more OTUs belonging to Prevotellaceae compared with Group C. Meanwhile, the number of OTUs belonging to Akkermansiaceae in Group PA+ was larger than that in Group PA− (*p* < 0.05; Supplementary Figure S9A). The treatments changed the variation rate of the relative abundance of Muribaculacceae along raising time. There was a significantly negative correlation with raising time in Group C, but no significant correlations were found in Groups PA+ and PA− (Supplementary Figure S9B). The treatments accelerated the decrement rate of the relative abundance of Ruminococceae. Specifically, a significantly negative correlation was observed between the relative abundance of Ruminococceae and raising time in Groups PA+ and PA− but not in Group C (Supplementary Figure S9B). Moreover, the treatments slowed down the reduction rate of the OTUs in Muribaculacceae and Prevotellaceae but accelerated the reduction rate of the OTUs in Ruminococceae. Meanwhile, the number of oocysts was positively correlated with the OTU number of Akkermansiaceae in Group PA− (*R*^2^ = 0.138, *p* = 0.025; Supplementary Figure S9B).

## 4 Discussion

### 4.1 Parasite-associated differences in gut bacterial communities

The gut microbiota structure commonly changes with interventions for parasitic infection (McKenney et al., 2015; Brosschot and Reynolds, 2018; White et al., 2018; Kupritz et al., 2021). However, our data showed no significant difference in gut bacterial structure among the treatments at each time stage but revealed significant *Eimeria* infection-induced changes in the host gut bacterial members. The first reason for this inconsistency may be associated with the host and parasitic species. Second, the present study is based on parasitic infection intensity rather than parasite colonization or absence of colonization in the host gut. The third reason may be related to the current study’s sample size. Although we have strived to conduct this study by using as many as possible samples, the available replicates were only 10 pikas in each treatment group. This small sample size may have influenced our results based on the random variations or specific characteristics of the individuals within the sample, leading to limited findings.

Parasite-modified changes in gut microbial members are generally related to shifts in microbial functions (Peachey et al., 2017) and react to parasites (Wang et al., 2023). For example, parasitic infections in mosquitoes (Culicidae) are characterized by some bacterial taxa that can biosynthesize ansamycin and vancomycin antibiotics and the pentose phosphate pathway (Trzebny et al., 2023). In our study, the highest relative abundance of the genus *Ruminococcus* was found in the treatment with anticoccidia, and the highest relative abundance of the species *Ruminococcus flavefaciens* was observed in the control group. The significantly varied OTUs in the treatments were mainly members of the family Muribaculaceae and enriched in the treatment involving *Eimeria* feeding. These differences in bacterial members in our study might be associated with parasite-modified energy metabolism patterns. The genus *Ruminococcus* is an important taxonomic rank that degrades and converts polysaccharides into nutrients (Reau and Suen, 2018). The members of Muribaculaceae are versatile in terms of complex carbohydrate degradation (Lagkouvardos et al., 2019) and generalists in terms of mucosal sugar utilization (Pereira et al., 2020). This feature indicates that the host might mobilize energy to respond to parasitic infections (Nadler et al., 2021) by altering bacterial communities.

Parasite-induced variations in gut microbial compositions are usually accompanied by differing diversities (Lee et al., 2014; Cortés et al., 2020a). Our results showed no significant changes in gut bacterial alpha diversity among the treatments over the course of the trial. Although the alpha diversity of microorganisms may change due to parasitic interference (Broadhurst et al., 2012; Cattadori et al., 2016; Lu et al., 2021), it may not always vary. Decrements in alpha diversity caused by parasitic infections were observed in some studies (Holm et al., 2015; Cattadori et al., 2016), whereas other studies reported elevated alpha diversity due to parasites (Broadhurst et al., 2012; Lee et al., 2014; Andersen and Stensvold, 2016). Consistent with our study, a few studies showed that gut microbial alpha diversities remain unchanged after parasitic infections (Kreisinger et al., 2015; McKenney et al., 2015; Li R. W. et al., 2016). These different gut microbial responses of hosts to parasites might be associated with the different host species and parasites (Barelli et al., 2021). However, the variable response patterns of the host gut microbe to parasitic infections are difficult to ascertain because of the complex interactions between gut microbiota and parasites. For example, while helminth infection might result in increased (Andersen and Stensvold, 2016), decreased (Cattadori et al., 2016), or unchanged (McKenney et al., 2015) gut microbial alpha diversity, so does coccidium infection (Macdonald et al., 2017; Lu et al., 2021). On the other hand, the different parasites might have different effects on the gut microbes of the same host species. For instance, the gut bacterial species observed in human children showed no change after *Cryptosporidium* infection but decreased after *Giardia intestinalis* infection and increased after *Ascaris* infection coinfected with *Cryptosporidium* or *Giardia intestinalis* (Toro-Londono et al., 2019).

Different from alpha diversity, beta diversity based on the Bray–Curtis distance of dissimilarity within groups was the highest in the control group and the lowest in the group with anticoccidia in the present study. This finding reflects the convergence of gut microbial communities caused by parasitic infections; that is, the parasites had a certain selective effect on gut microbiota. These certain selections might be associated with the increase in selected microbial taxa (Kreisinger et al., 2015) or variations in the specific function of microbiota (Wang et al., 2023). Our data revealed a certain selection of a few parasite-associated shifting bacterial members that might be associated with energy metabolism. However, the results could not determine the specific development direction of the host gut microbe induced by parasites.

### 4.2 Parasite-modified patterns of temporal alterations in gut bacterial communities

Our results showed that the raising time obviously had effects on the gut bacterial community, which overwhelmed the parasitic impact. Given the remarkable dietary plasticity of gut microbes (Leeming et al., 2019; Fan et al., 2022), the patterns in our results might be due to the change in diet from diverse plants in the field to simple feedstuff with low dietary fiber in the laboratory. Gut microbial diversity can be reduced dramatically by low-dietary fiber, which is widely recognized as a microbiota-accessible carbohydrate (Sonnenburg et al., 2016). Correspondingly, the gut bacterial alpha diversities declined significantly along raising time in our study.

Moreover, parasite infection changed the patterns of temporal variations in the gut bacterial communities. Our results showed that parasitic infection decreased the reduction rate of bacterial alpha diversity along laboratory raising time. This result echoes the prevalence of significantly positive correlations between oocyst number and the relative abundance of bacterial OTUs. In general, an increase in the alpha diversity of a gut microbial community signifies healthy intestinal homeostasis (Clemente et al., 2012). Thus, the reduced rate patterns in our study might indicate healthy pika intestines modified by coccidia.

*Eimeria* disturbs the temporal model of bacterial members. Parasites might exert a selective effect on specific microbial taxa to defend against infection damage (Brosschot and Reynolds, 2018; Gao et al., 2020) or enhance the parasitic advantage (Reynolds et al., 2014). For example, a study found that *Babeisa duncani* infection increases the relative abundance of the family Muribaculaceae (Zhang et al., 2023). Similarly, in our study, the *Eimeria* infections reduced the loss rate of the relative abundance and OTU number of the family Muribaculaceae. A reduced loss rate due to *Eimeria* was observed in the family Prevotellaceae, but an accelerated loss rate was found in Ruminococcaceae. The members of Prevotellaceae can produce butyrate (Chen et al., 2022), so they play important roles in intestinal homeostasis and inflammation regulation (Wang et al., 2020). Ruminococcaceae members are fibrolytic specialists (Biddle et al., 2013). Thus, parasite-associated temporal models of bacterial members might be associated with the intestinal homeostasis of pikas and the gut energy metabolite after *Eimeria* infections.

### 4.3 Tripartite relationships among parasites, microbes, and the host

Aside from the microbe-associated energy strategy of pikas after parasitic infections, we also observed a behavioral and physiological energy trade-off. The links of parasites with host behaviors and physiology are essential for understanding parasitic infection (Escobedo et al., 2005; Hughes and Libersat, 2019). In this study, the behavior and physiology of pikas were modified by *Eimeria* feeding and anticoccidia treatments. The thyroid hormones (T3 and T4) were increased by *Eimeria* feeding, whereas exploration activity decreased. The increased thyroid hormone in the group with *Eimeria* feeding suggested high energy metabolism (Speakman et al., 2021) because of the parasite-induced energy costs (Slavík et al., 2017). The reduced exploration activity might be due to a trade-off in the energy allocation of hosts after parasite infection. This statement is supported by the increase in thyroid hormone and the decrease in exploration activity with increasing time in the group with anticoccidia.

The tripartite correlation among animals, gut microbiota, and gut parasites is important to the health of animals after parasitic infection (Stensvold and Giezen, 2018; Madlala et al., 2021). Our results showed notable links between the bacterial community, behavior, and physiology. We also found a parasite-associated energy strategy with alterations in the behavior, physiology, and gut bacteria of taxa members. However, how gut bacterial communities specifically relate to pika behaviors and physiology as specific responses to parasitic infection remain unclear. We could not determine the role of gut bacteria in the energy allocation between parasites and the host. Specifically, we could not ascertain whether gut bacteria benefit the parasites or the host.

## 5 Conclusion

This study focused on parasite-associated alterations in gut bacterial communities, host behaviors, and host physiology, as well as host–bacteria relationships. The use of Bray–Curtis distance metrics revealed insignificant differences in bacterial community structure among the treatments, but notable alterations were observed along raising time in the laboratory. The alpha diversities did not vary with the treatments but were considerably reduced as time increased. Moreover, experimental feeding with *Eimeria* maintained the bacterial alpha diversities of the pika gut with a reduced decrement rate of alpha diversity. A few bacterial members exhibited notable shifting. Only the genus *Ruminococcus*, the species *Ruminococcus flavefaciens*, and several OTUs that belonged to the family Muribaculaceae were associated with energy metabolism. Notably, the temporal variations in the bacterial members were altered by parasitic infection. For instance, experimental feeding with *Eimeria* reduced the loss rate of the relative abundance of the dominant families, Muribaculaceae and Ruminococcaceae. Meanwhile, the parasite-associated variations in pika behavior and physiology showed an energy trade-off after *Eimeria* infection. However, no specific energy-associated connections were found between gut bacteria and host behavior or physiology. Our findings highlight the potential implications of the temporal responses of host gut bacterial communities underpinning host–parasite interactions. However, we did not find a certain energy trade-off in the relationships of gut bacterial communities with pika physiology and behavior. We will conduct further in-depth research in the future to address these questions.

## Data availability statement

The datasets presented in this study can be found in online repositories. The names of the repository/repositories and accession number(s) can be found in the article/supplementary material.

## Ethics statement

The animal study was approved by Ethics Committee, Northwest Institute of Plateau Biology, Chinese Academy of Sciences. The study was conducted in accordance with the local legislation and institutional requirements.

## Author contributions

ML: Writing – original draft, Writing – review & editing. SW: Investigation, Writing – review & editing. LZ: Writing – review & editing. PH: Writing – review & editing. ZT: Writing – review & editing. RW: Writing – review & editing. XC: Writing – review & editing. YZ: Writing – review & editing. BT: Writing – review & editing. HZ: Writing – review & editing. JQ: Funding acquisition, Project administration, Writing – review & editing.
