# Supplementary material for: Eimeria infections of plateau pika altered the patterns of temporal alterations in gut bacterial communities
Source: Front Microbiol. 2024 Jan 11;14:1301480. doi: 10.3389/fmicb.2023.1301480 (PMC10808676; doi:10.3389/fmicb.2023.1301480)
Supplement: Supplementary file 1 [file Data_Sheet_1.pdf]

Supporting information

Eimeria infections of plateau pika altered the patterns of temporal alterations in gut bacterial communities

Maoping Li[1,2#], Suqin Wang[1,3#], Liang Zhong[1,2], Petr Heděnec[4], Zhaoxian Tan[1,5], Rong Wang[1,5], Xinyang Chen[1,3], Yan Zhang[1,3], Bingmin Tang[6], Huakun Zhou[1], Jiapeng Qu[1,2]*

[1]Sanjiangyuan Grassland Ecosystem National Observation and Research Station, Key Laboratory of Adaptation and Evolution of Plateau Biota, Northwest Institute of Plateau Biology, Chinese Academy of Sciences, Xining, 810008, China

[2]Qinghai Province Key Laboratory of Animal Ecological Genomics, Xining, 810008, China

[3]University of Chinese Academy of Sciences, Beijing, 100049, China

[4]Institute for Tropical Biodiversity and Sustainable Development, University Malaysia Terengganu, 21030, Kuala Nerus, Terengganu, Malaysia

[5]School of life Science, Qinghai Normal University, Xining, 810008, China

[6]Grassland Station of Qinghai Province, Xining, 810008, China

# These two authors contribute equally to this work.

* Correspondence: jpqu@nwipb.cas.cn; Tel.: +86 13997063739

Supplemental files：

Table S1
Figure S1-S9

Table S1. The correlations between driving factors and bacterial communities using envfit analysis

Factors	All		PA+		C		PA-	
	r	p	r	p	r	p	r	p
Oocysts number	**0.120**	**0.031**	**0.324**	**0.005**	0.014	0.238	0.075	0.105
Cortisol	0.060	0.548	**0.304**	**0.005**	0.103	0.065	0.058	0.184
T3	0.030	0.872	0.103	0.113	**0.181**	**0.001**	0.072	0.100
T4	0.100	0.144	**0.474**	**0.001**	0.048	0.104	0.083	0.077
RMR	**0.200**	**0.003**	0.093	0.123	**0.298**	**0.047**	**0.331**	**0.003**
Exploration	0.020	0.958	**0.349**	**0.044**	0.020	0.118	**0.287**	**0.021**
Weight	0.030	0.261	0.070	0.180	**0.300**	**0.040**	**0.151**	**0.019**
Treatment	**0.140**	**0.021**	--	--		--		--
Time	--	--	0.051	0.268	**0.308**	**0.001**	**0.115**	**0.034**

Significant results are highlighted in bold letters

A

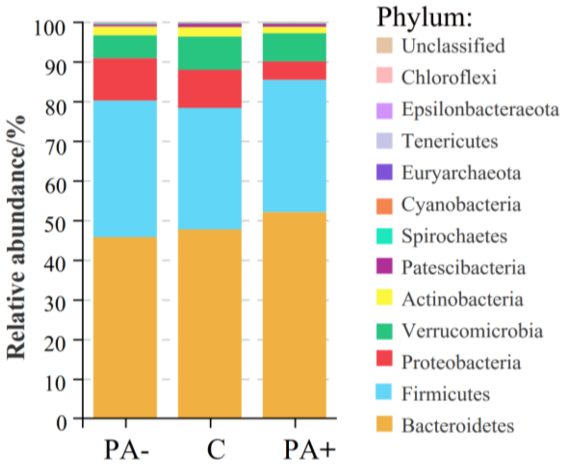

B

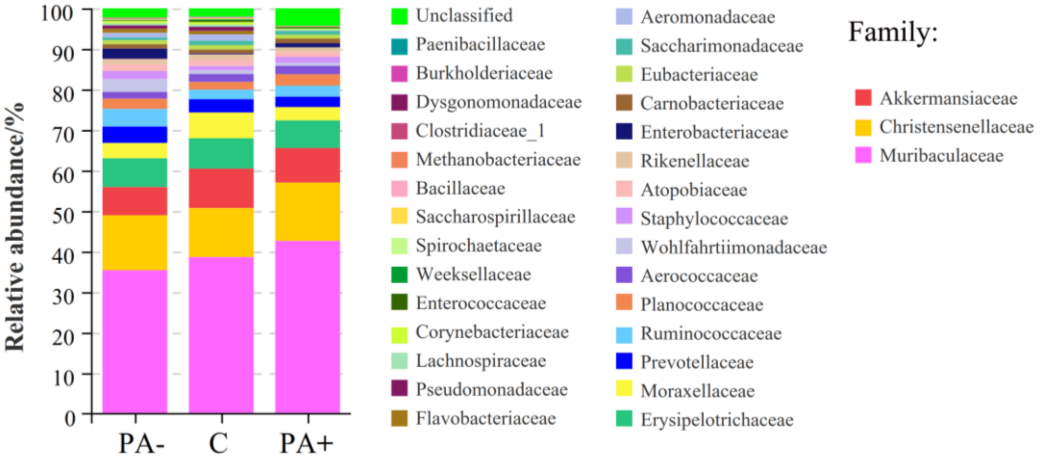

C

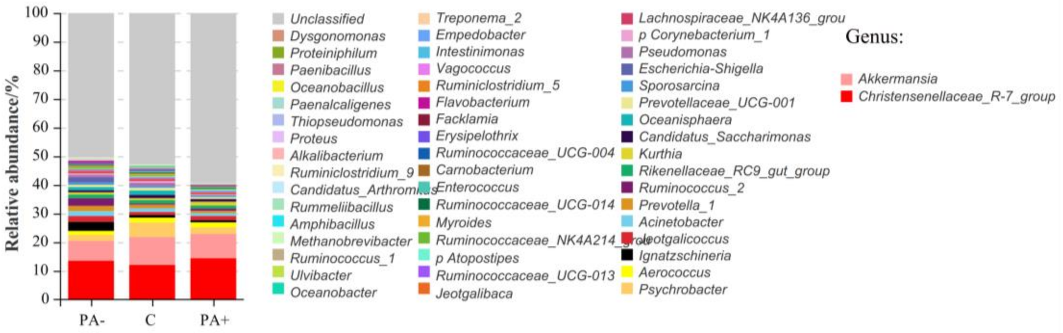

Figure S1. Bacterial community compositions across all samples at (A) phylum, (B) family and (C) genus level. Only those phyla with mean relative abundance > 0.1% and family with mean relative abundance > 1%, as well as genus with mean relative abundance > 1% are shown

A

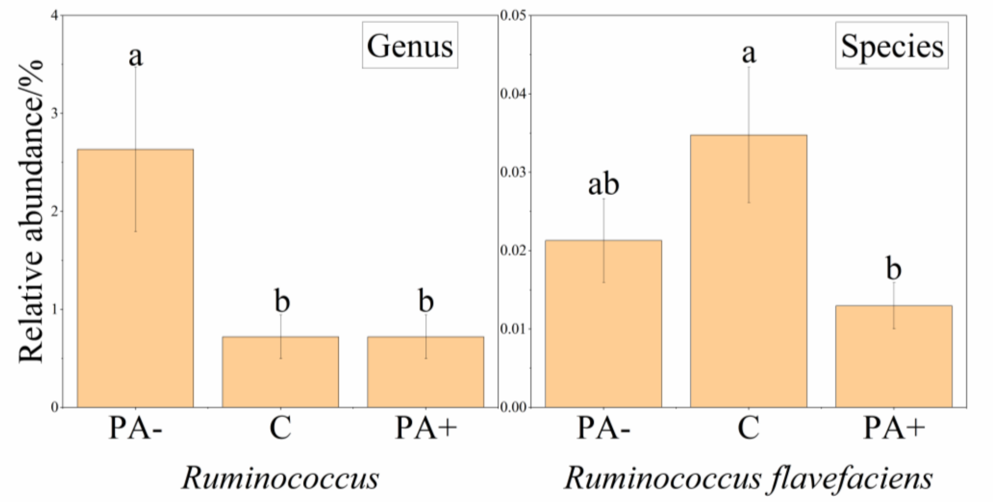

B

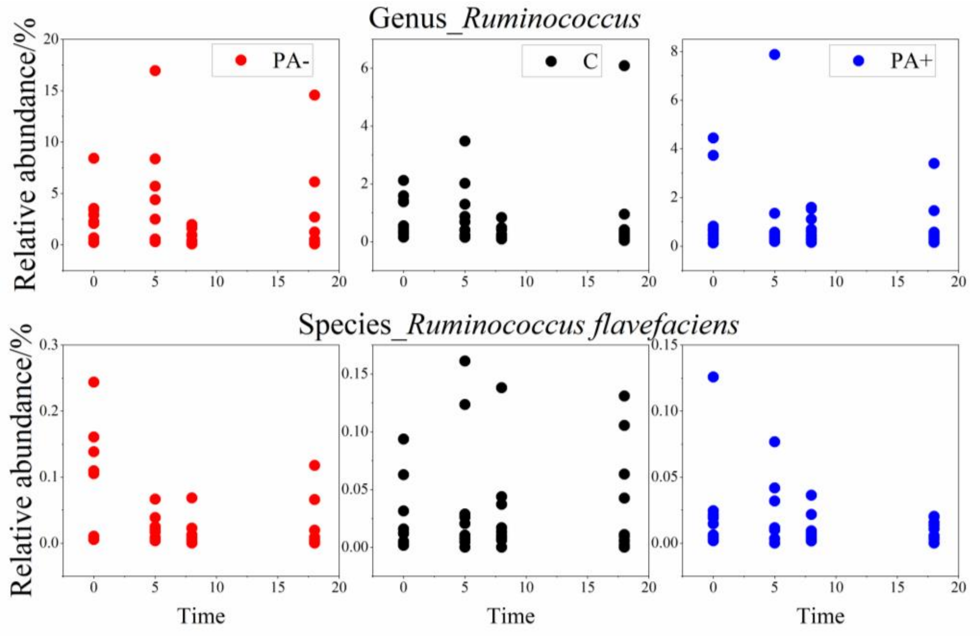

Figure S2. (A) The variations in the bacterial taxa of pika gut bacterial communities at genus and specie level among treatments. Different lower-case letters indicate a significant difference at $p < 0.05$. (B) The correlations between time and the relative abundance of bacterial genus and species which were significantly shifted by treatments

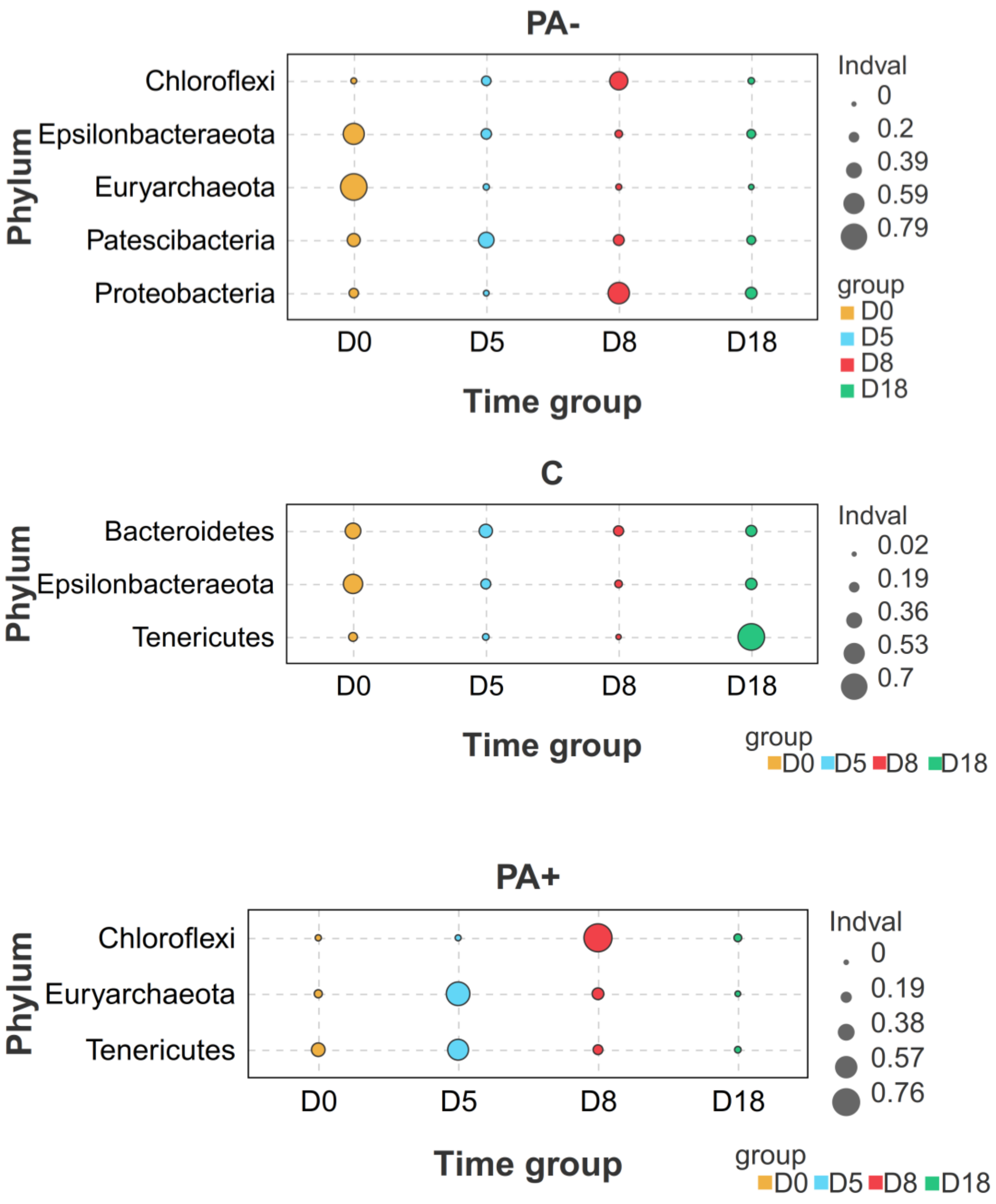

Figure S3. The indicator taxa calculated using species indicator analyses at each time stage at phylum level ($P < 0.05$) of each treatment group

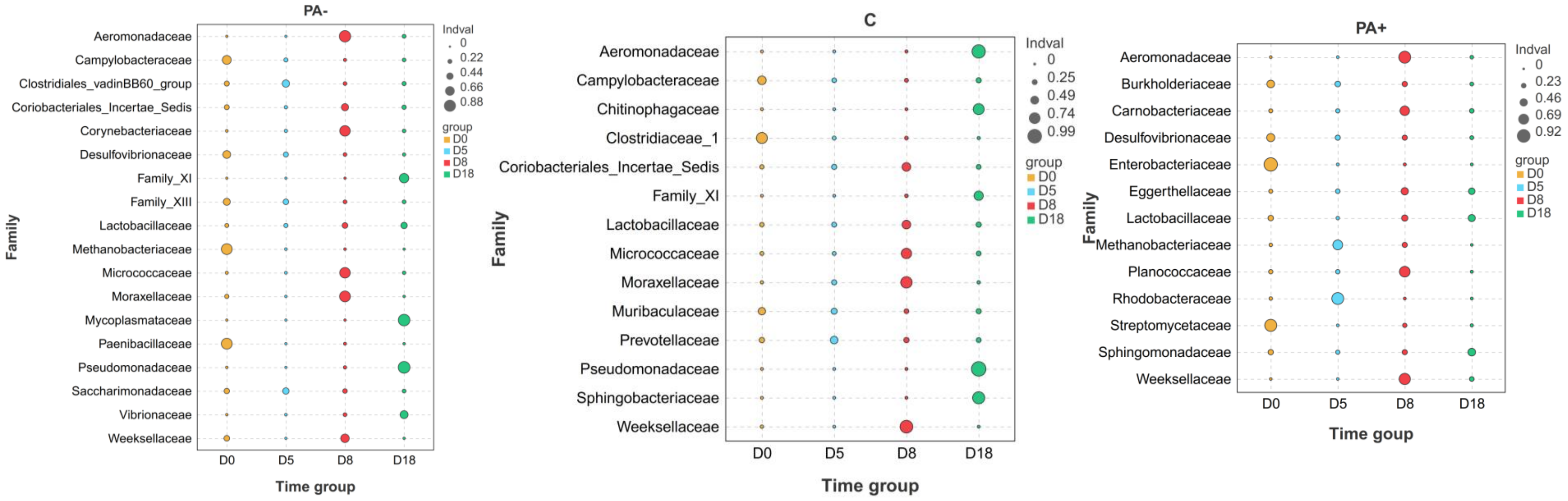

Figure S4. The indicator taxa calculated using species indicator analyses at each time stage at family level ($P < 0.05$) of each treatment group

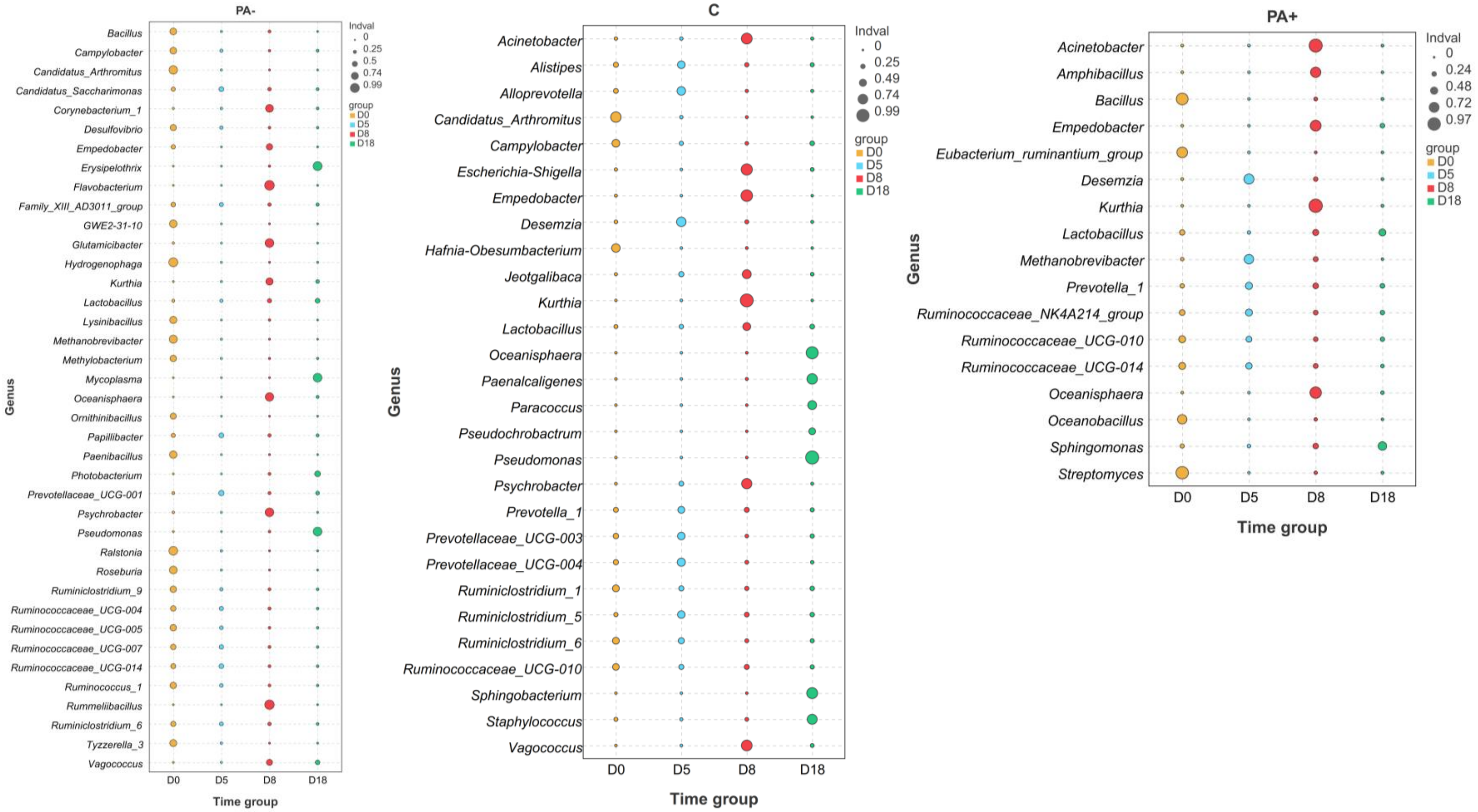

Figure S5. The indicator taxa calculated using species indicator analyses at each time stage at genus level ($P < 0.05$) of each treatment group

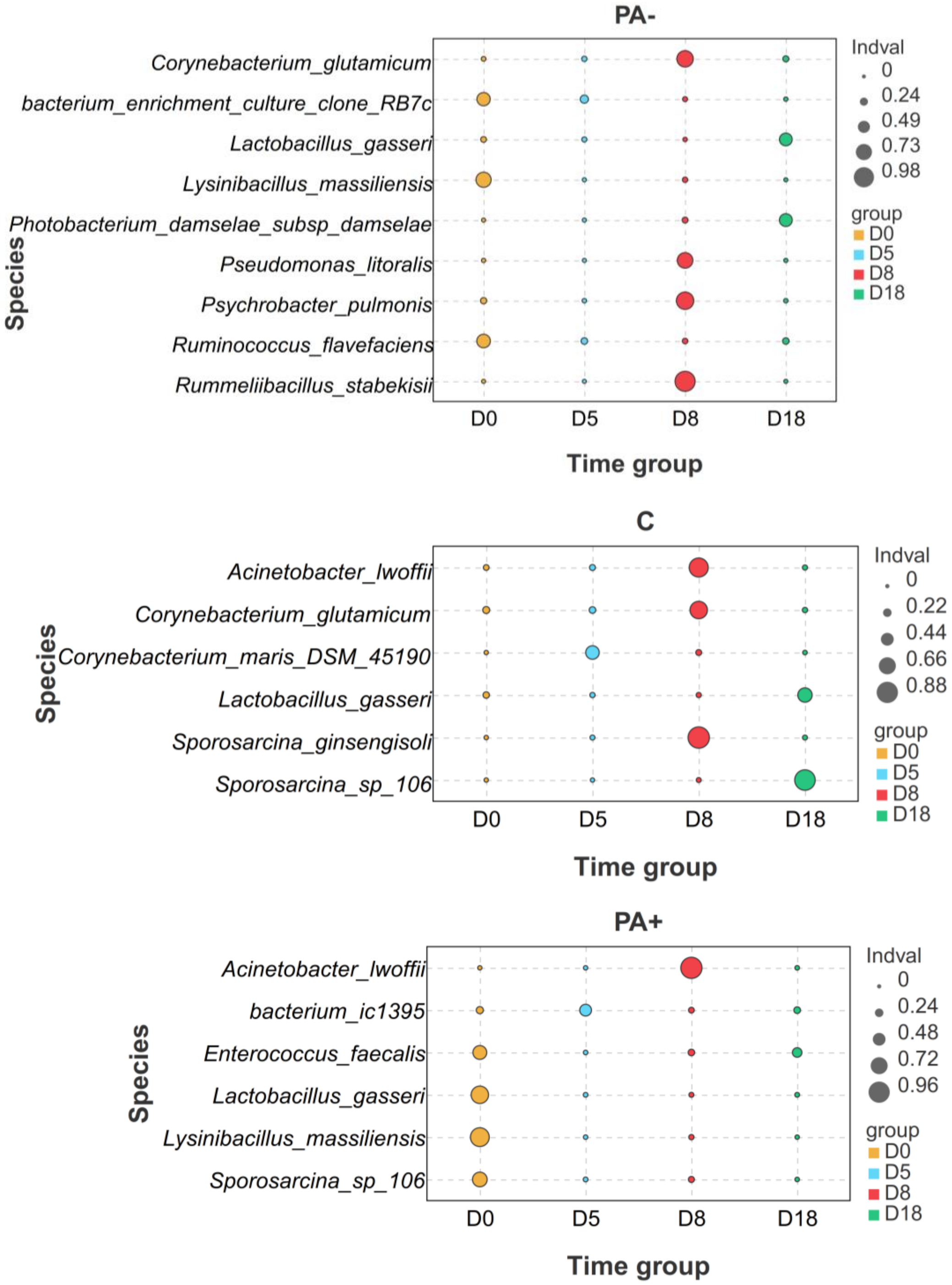

Figure S6. The indicator taxa calculated using species indicator analyses at each time stage at species level ($P < 0.05$) of each treatment group

A

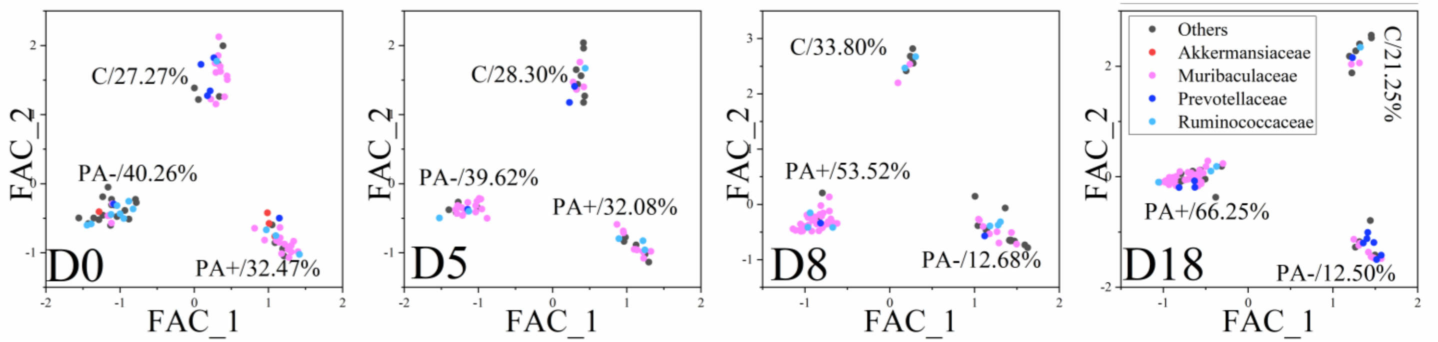

B

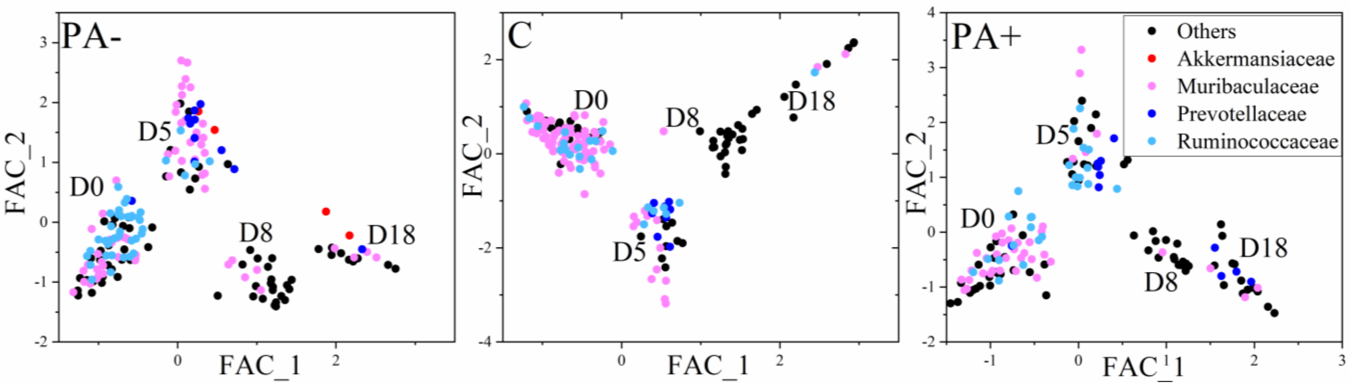

Figure S7. The indicator OTUs calculated using species indicator analyses A)among treatments ($P < 0.05$) at each time stage and B)among each time stage ($P < 0.05$) of each treatment group

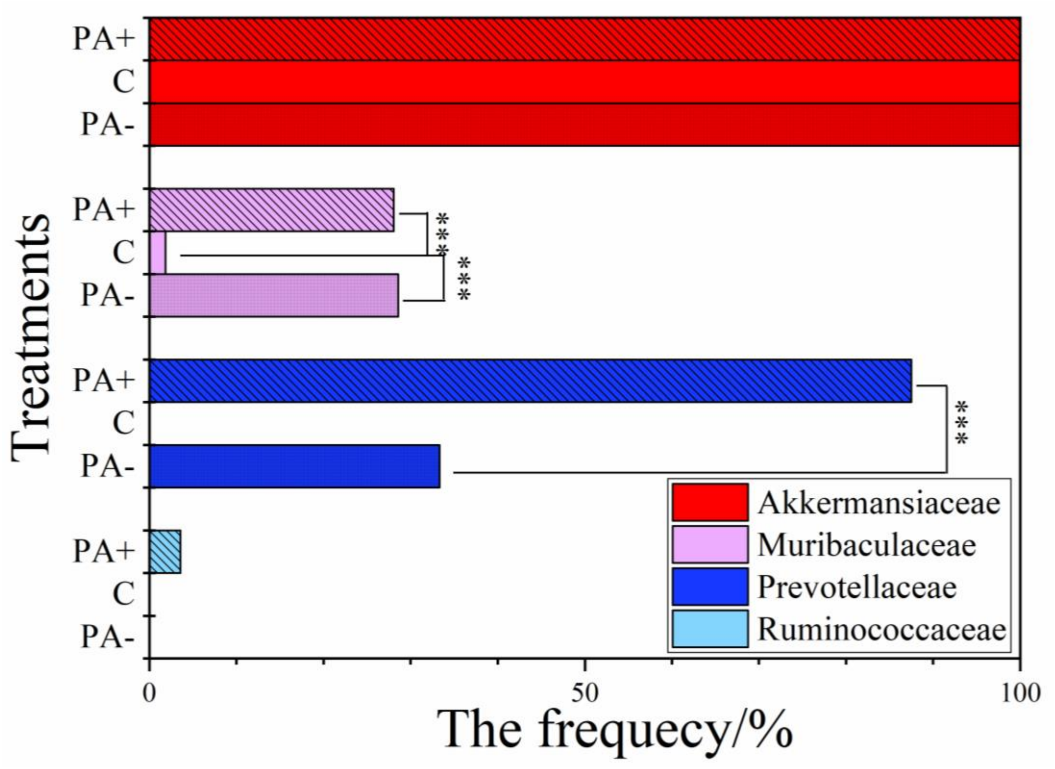

Figure S8. The variations in the frequency of significantly positive correlations between time and the relative abundance of bacterial OTUs among treatment groups

A

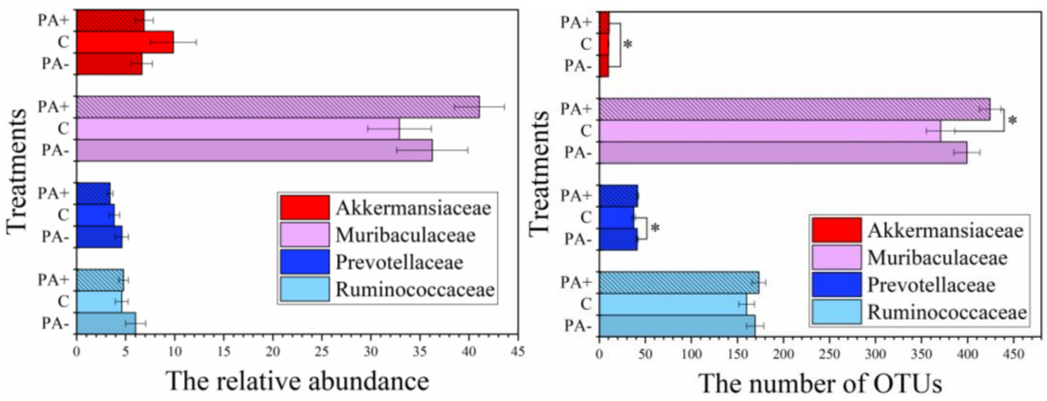

B

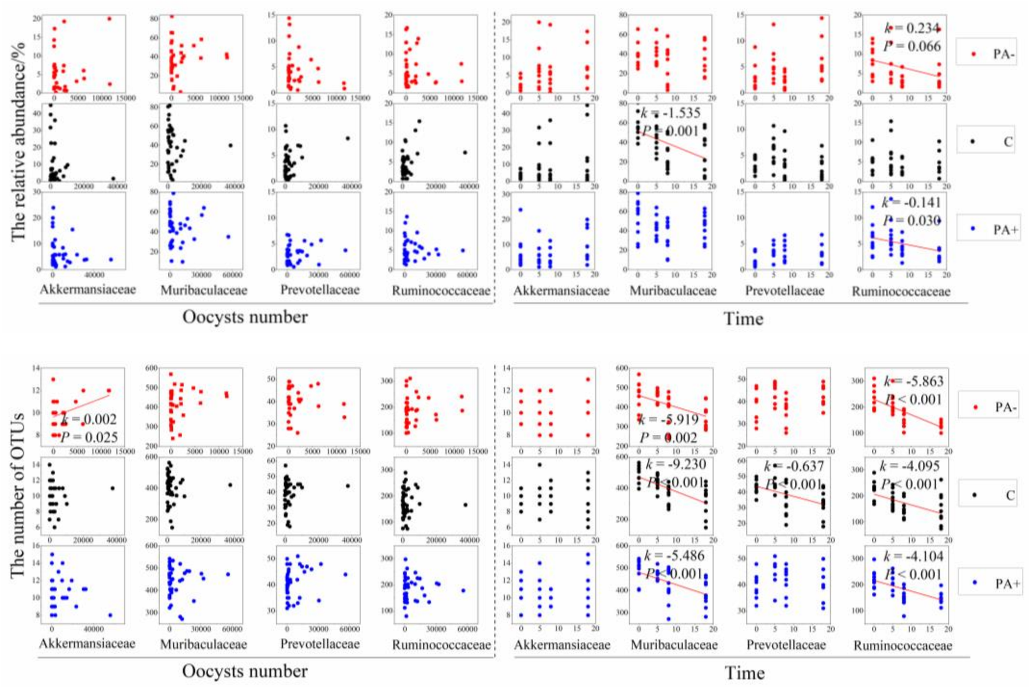

Figure S9. (A) The variations in the number of OTUs and relative abundance of the bacterial families Akkermansiaceae, Muribaculaceae, Prevotellaceae and Ruminococcaceae among treatments, (B) The correlations of oocysts number and raising time with the number of OTUs and relative abundance of the bacterial family Akkermansiaceae, Muribaculaceae, Prevotellaceae and Ruminococcaceae.
